# Rivaroxaban improves vascular response in LPS-induced acute inflammation in experimental models

**DOI:** 10.1371/journal.pone.0240669

**Published:** 2020-12-10

**Authors:** Armond Daci, Lorenzo Da Dalt, Rame Alaj, Shpejtim Shurdhiqi, Burim Neziri, Rrahman Ferizi, Giuseppe Danilo Norata, Shaip Krasniqi

**Affiliations:** 1 Department of Pharmacy, Faculty of Medicine, University of Prishtina, Prishtina, Kosovo; 2 Institute of Pharmacology and Toxicology, Faculty of Medicine, University of Prishtina, Prishtina, Kosovo; 3 Department of Excellence of Pharmacological and Biomolecular Sciences, Università degli Studi di Milano, Milan, Italy; 4 Cardiovascular Surgery Clinic, University Clinical Center of Kosovo, Prishtina, Kosovo; 5 Institute of Pathophysiology, Faculty of Medicine, University of Prishtina, Prishtina, Kosovo; 6 Department of Premedical Courses-Biology, Faculty of Medicine, University of Prishtina, Prishtina, Kosovo; 7 Centro SISA per lo Studio dell’Aterosclerosi, Ospedale Bassini, Cinisello Balsamo, Italy; Nagoya University, JAPAN

## Abstract

Rivaroxaban (RVX) was suggested to possess anti-inflammatory and vascular tone modulatory effects. The goal of this study was to investigate whether RVX impacts lipopolysaccharide (LPS)-induced acute vascular inflammatory response. Male rats were treated with 5 mg/kg RVX (oral gavage) followed by 10 mg/kg LPS i.p injection. Circulating levels of IL-6, MCP-1, VCAM-1, and ICAM-1 were measured in plasma 6 and 24 hours after LPS injection, while isolated aorta was used for gene expression analysis, immunohistochemistry, and vascular tone evaluation. RVX pre-treatment significantly reduced LPS mediated increase after 6h and 24h for IL-6 (4.4±2.2 and 2.8±1.7 fold), MCP-1 (1.4±1.5 and 1.3±1.4 fold) VCAM-1 (1.8±2.0 and 1.7±2.1 fold). A similar trend was observed in the aorta for iNOS (5.5±3.3 and 3.3±1.9 folds reduction, P<0.01 and P<0.001, respectively), VCAM-1 (1.3±1.2 and 1.4±1.3 fold reduction, P<0.05), and MCP-1 (3.9±2.2 and 1.9±1.6 fold reduction, P<0.01). Moreover, RVX pre-treatment, improved LPS-induced PE contractile dysfunction in aortic rings (Control *vs* LPS, Emax reduction = 35.4 and 31.19%, P<0.001; Control *vs* LPS+RVX, Emax reduction = 10.83 and 11.48%, P>0.05, respectively), resulting in 24.5% and 19.7% change in maximal constriction in LPS and LPS+RVX respectively. These data indicate that RVX pre-treatment attenuates LPS-induced acute vascular inflammation and contractile dysfunction.

## Introduction

Coagulation plays a key role in cardiovascular disorders [1] and interfering with coagulation factors represents one of the main pharmacological approaches in CVD [2]. Coagulation factors, not only participate to the activation of the coagulation cascade but also impact vascular function; this is the case for factor X (FXa), one of the main components in the coagulation process [3], which, through the activation of protease-activated receptors (PAR) [4], affects, vasomotor responses, inflammation, endothelial function, vascular proliferation, cellular hypertrophy, atherosclerosis, and thrombosis [5,6].

As hemostatic and inflammatory pathways are highly interconnected [7,8], the approval of novel oral anticoagulants (NOAC), affecting FXa activity and prothrombin complexes (rivaroxaban, apixaban, betrixaban, and edoxaban) [9], have raised interest in the interplay between haemostasis and inflammation linking FXa blockade to PAR inhibition [10,11], and potentially to improved vascular function.

Clinical studies with NOACS have shown that these drugs reduce the incidence of cardiovascular events including coronary and peripheral artery disease, cerebral ischemia, thrombosis, thromboembolic events, and atherosclerosis [12,13]. In addition to this, experimental studies proposed a series of vascular protective properties of NOAC via inhibition of FXa [14–26]. These include potential anti-inflammatory effects [15,19–21,23,24,27], that might perhaps impact vascular function and pathology [14,25]. Indeed inflammation is one of the main contributing factors in coronary artery disease leading to the development of atherosclerosis [28]. Moreover, acute exposure to lipopolysaccharide endotoxin (LPS) has been shown to induce an inflammatory response that in turn supports vascular injury and dysfunction [29,30].

This raises the intriguing possibility that the impact on NOACS on vasomotor function [17,31] might depend also on the ability to control vascular function under acute inflammatory conditions.

To this aim, we used isolated rat aorta, to test the hypothesis that pre-treatment with Rivaroxaban (RVX) might mitigate LPS-induced acute vascular inflammation with a focus on pro-inflammatory, pro-adhesive, and contractile responses under LPS-induced vascular inflammatory conditions.

## Material and methods

### Animals and treatment

Wistar rats between 10–12 weeks of age (220–260 g) were used in our study. All rats were fed with a normal chow diet during the period of our study. Animals were accommodated in normal rat cages with automatically controlled 12-hours light/12-hour dark cycle and the standard temperature-humidity environment with ad libitum water and food intake. Acute inflammation was induced by a single intraperitoneal (i.p) injection of LPS (10 mg/kg body weight) [32]. RVX (5 mg/kg body weight; supplied by Bayer Pharma AG) was administered via oral gavage 2 hours before LPS injection, the non-RVX groups (control and LPS only) received oral gavage of RVX vehicle (Carboxymethylcellulose Sodium 0.5%). RVX dose and interval used in our study was previously shown to inhibit the in vivo Factor Xa in rat arteriovenous shunt model [33] or thrombus formation [34] and was chosen based on previous in-vivo related mice and rat animal studies [14,17,35,36], which are specific in the previously reported single p.o administration of the RVX pharmacokinetic profile as well [37,38]. Our study protocol has been approved by the Ethical Committee of Medical Faculty–University of Prishtina (Nr. 4962), and all procedures for animal experiments were performed in compliance with guidelines for care and use of animals during whole experimentation procedures.

## ELISA

Rats were sacrificed with an i.p overdose of sodium thiopental injection (50 mg/kg body weight) at 6 hours and 24 hours after LPS injection. Blood was collected from the left ventricle (EDTA containing tubes) and plasma isolated following centrifugation (4000 rpm for 10 minutes) and stored at -80°C. IL-6, MCP-1, VCAM-1, and ICAM-1 plasma levels were measured by enzyme-linked immunoassay kit (Abcam, Cambridge, MA) according to manufacturer’s protocol instructions.

### Aorta preparation

After blood collection, the whole rat aorta was isolated and cleaned immediately from adhering perivascular adipose and connective tissues. Aortic rings of 4–6 mm were cut from the ascending aorta and fixed in 10% neutral buffered formalin for further immunohistochemical analysis. Subsequently, aortic rings of 5–7 mm were cut from the remaining part of the ascending aorta and descending aorta and snap-frozen in liquid nitrogen for gene expression analysis. Finally, aortic rings of 3–5 mm were cut from the remaining part of the thoracic aorta and used for testing vascular reactivity in the tissue organ bath.

### Immunohistochemistry

Formalin-fixed aortic rings were embedded in paraffin and sectioned in 2.5 μm sections. Tissue sections were deparaffinized as described [39], rehydrated and the heat mediated antigens retrieval was performed by placing the slides in 10 mM sodium citrate buffer (pH 6.0) for 45 minutes at 95–98°C. Blocking of endogenous peroxidase activity and non-specific staining was done with hydrogen peroxide and protein block. Subsequently, sections were incubated for 30 minutes with primary antibodies at the following dilution: 1:200 for anti-VCAM-1, 1:200 for anti-MCP-1, or 1:100 for Anti-iNOS. After washing steps, sections were incubated with a biotinylated secondary antibody (goat anti-polyvalent, Mouse, and Rabbit Specific HRP/DAB (ABC) Detection IHC kit, Abcam, Cambridge MA) for additional 15 minutes followed with streptavidin peroxidase 10’ incubation. Peroxidase activity was detected in fixed tissues with DAB substrate chromogen for detection of HRP-conjugated antibody and followed under a microscope to determine staining development. Finally, after the tipping DAB and rinsing in water, the slides were counterstained with hematoxylin histological staining reagent as described [40]. Images were acquired with Olympus CX41 microscope (Olympus America) with Olympus SC100 Digital camera and cell Sens Imaging Software. Relative quantification of IHC staining has been done with Image J (NIH, https://imagej.nih.gov/ij/).

### Reverse transcription-quantitative real-time PCR (RT-qPCR) analysis

Total RNA was isolated from aortae by using RNeasy Fibrous Tissue Mini Kit (Qiagen, Hilden, Germany) following the standard protocol. RNA assessed for quality and quantity using absorption measurements (NanoDrop™ 1000 Spectrophotometer, Thermo Fisher Scientific) and retro-transcribed in cDNA with iScriptTM cDNA synthesis kit (BioRad) as described [41]. Gene expression analysis was performed using SYBR Green Supermix (Thermo Fisher Scientific) in CFX connect light cycler (BioRad, Cat#1708841) [42]. Expression was calculated using the ΔΔCt method (Livak and Schmittgen, 2001) and normalized to a housekeeping gene (GAPDH). The sequences are presented in S1 Table and expression levels were expressed with the fold change.

### Vascular tone

Aortic ring fragments were mounted in the Tissue Organ Baths (750TOBS, DMT-USA, Ann Arbor, MI, USA) containing 10 mL of Krebs-Henseleit buffer (118.4 mM NaCl, 4.7 mM KCl, 2.5 mM CaCl2, 1.2 mM KH2PO4, 1.2 mM MgSO4, 25 mM NaHCO3, 11.1 mM glucose; pH 7.4). The temperature was adjusted at 37°C and the buffer solution was bubble gassed with 5% CO2 and 95% O2 during the whole experiment. Changes in force tension were recorded by isometric force-displacement transducers and continuous mode on a multichannel recorder polygraph model attached with software LabChart7 connected to power lab 4/35 data acquisition system (PowerLab 4/35, ADInstruments Pty Ltd., NSW, Australia).

Each ring was initially stretched to an optimal load (∼2 g). Subsequently, preparations were equilibrated for 60 minutes with changes of fluid every 15 minutes. After the equilibration period, vessel specimen viability was tested with KCl (40 mM) induced contraction, and aortic segment preparations were washed until returning the basal tone. Thereafter, the vascular tone was determined with cumulative concentration-response curves with phenylephrine (10^−8^ to 10^−5^ M).

### Statistical analysis

All data are presented and calculated with mean ± SEM. The number of rats used in our study was expressed with “n”. A comparison of parameters obtained within the analysis between two groups was performed with unpaired Student’s t-test. Vasoreactivity of the PE contractions was calculated as a percentage of the KCl (40 mM) maximal initial contraction value. The concentration-response curve values were analyzed with two-way ANOVA followed by Tukey’s post hoc test for comparison between groups. P-value < 0.05 was considered to represent a statistically significant group difference. All analyses and graphs were performed using GraphPad PRISM version (6.0).

## Results

### 3.1 Effects of RVX pre-treatment on the acute LPS-induced increase in IL-6, MCP-1, VCAM-1 and ICAM-1 levels

**Fig 1:**
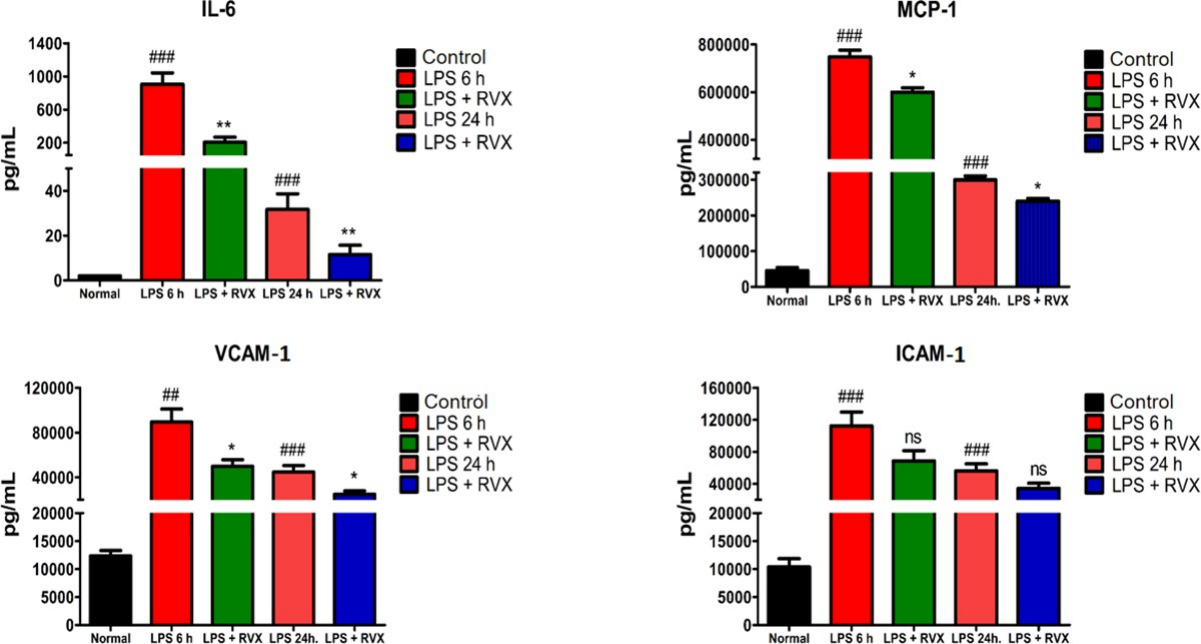
Role of RVX pre-treatment on LPS-induced proinflammatory and proadhesive mediator release in rat plasma. IL-6 (A), MCP-1 (1B), VCAM-1 (C) and ICAM-1 (D) plasma levels (pg/mL) from LPS treated rats in the presence or absence of RVX for 6 hours and 24 hours compared to controls. ##P<0.01 and ###P<0.001 (Student’s t-test) vs. control conditions. *P<0.05, ** P<0.01 and ***P<0.001 (Student’s t-test) vs LPS. Values are expressed as the mean±SEM (n = 6).

In order to investigate the impact of RVX pre-treatment on LPS-induced pro-inflammatory and proadhesive mediators expression, IL-6, MCP-1, VCAM-1, and ICAM-1 levels were measured in plasma 6 hours and 24 hours post LPS injection. As expected, LPS injection increased plasma levels IL-6, MCP-1, VCAM-1, and ICAM-1(P<0.001) **(Fig 1A–1D)**; an effect which was significantly blunted with RVX pre-treatment (5 mg/kg) for IL-6 (P<0.01), MCP-1 (P<0.05) and VCAM-1 (P<0.05) both after 6 and 24 hours of treatment. These results suggest that RVX pretreatment limits LPS induced inflammatory response **(Fig 1A–1C).**

### 3.2 Effects of RVX pre-treatment on IL-6, MCP-1, VCAM-1 and ICAM-1 gene expression the aorta

**Fig 2:**
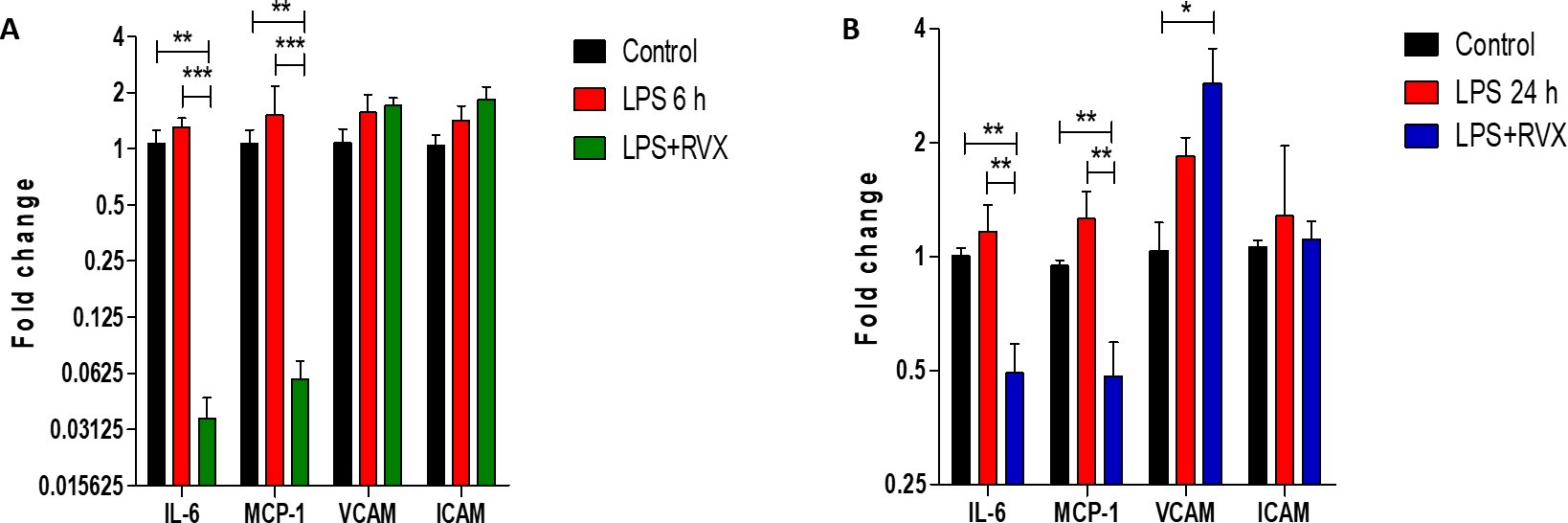
Role of RVX pre-treatment on LPS induced proinflammatory and proadhesive mediator’s gene expression in rat aorta. Comparisons of relative IL-6, MCP-1, VCAM-1 and ICAM-1 gene expression levels normalized to GAPDH in the rat aorta obtained from RVX- or non-treated LPS rats and non-treated control rats 6 hours (A) and 24 hours (B) post LPS exposure. *P<0.05, ** P<0.01 and ***P<0.001 indicates values significantly different (Student’s t-test) vs LPS. Values are expressed as the mean±SEM (n = 6).

Next, we tested whether RVX treatment could improve pro-inflammatory gene expression in the aorta and liver. RVX pre-treatment (5 mg/kg) attenuated IL-6 and MCP-1 mRNA expression in the arterial wall **(Fig 2A and 2B),** while non-significant changes were observed for VCAM-1 and ICAM-1 expression at this site. Most importantly RVX pre-treatment did not affect LPS induced liver expression of IL-6, MCP-1, VCAM-1, and ICAM-1, by confirming also the liver as a key target of LPS induced acute inflammation **(S1 Fig)**.

### 3.3 Effects of RVX pre-treatment on LPS-induced iNOS, MCP-1, and VCAM-1 wall expression in the vascular wall

**Fig 3:**
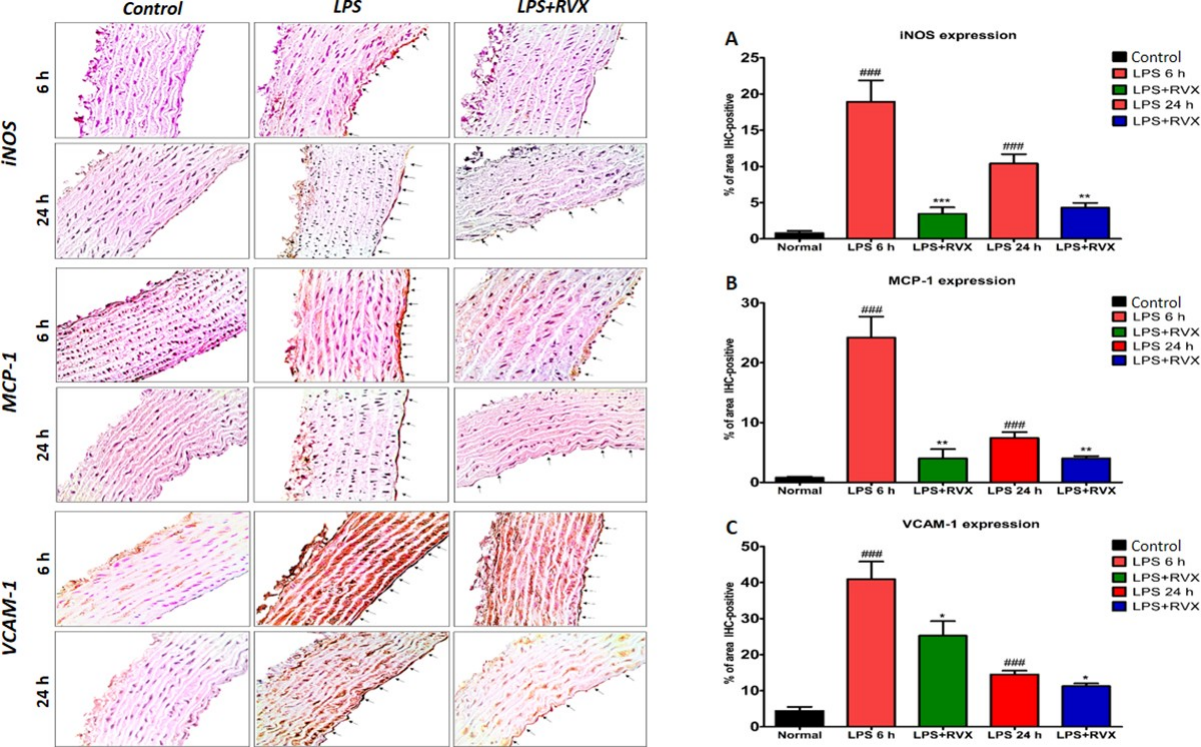
Role of RVX pre-treatment on LPS induced proinflammatory and proadhesive mediator’s protein expression in rat aorta. Representative immunohistochemical results for aortic iNOS (A), MCP-1 (B) and VCAM-1 (C) expressions from RVX- or non-treated LPS rats and non-treated control rats as described in the legend. % of IHC positive areas are represented as graphs. ### indicates values significantly different (Student’s t-test) vs. control conditions. *P<0.05, ** P<0.01 and ***P<0.001 indicates values significantly different (Student’s t-test) vs LPS. Values are expressed as the mean±SEM (n = 6).

Increased iNOS, MCP-1, and VCAM-1 immunoreactivity were observed in the aortic vascular tissues of LPS treated rats mainly in vascular endothelium, and the subendothelial layer was characterized by smooth muscle cells and perivascular adipose tissues compared to controls (P<0.001) **(Fig 3A–3C)**. RVX pre-treatment (5 mg/kg) reduced LPS-induced iNOS, MCP-1, and VCAM-1 expression both at 6 h and 24 h following LPS injection **(Fig 3A–3C).**

### 3.4 Effects of RVX pre-treatment on the acute LPS-induced PE contractile dysfunction in aortic rings

**Fig 4:**
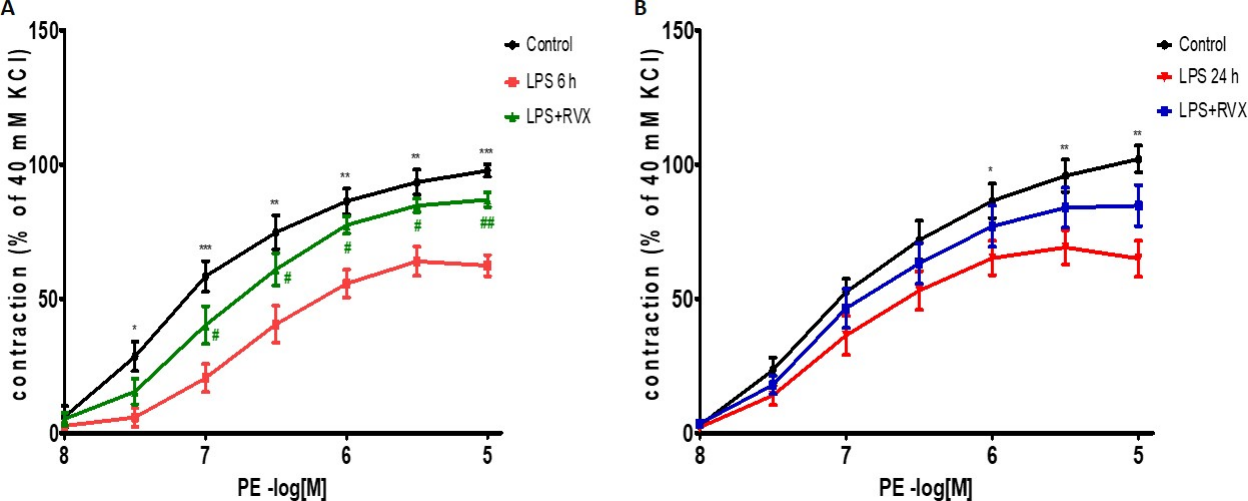
Role of RVX on the vasoreactivity of aortic rings obtained from A) rats sacrificed 6 hours post LPS and B) rats sacrificed 24 hours post LPS to PE-induced contractions. Comparisons of vascular reactivity to PE in aortic rings from RVX- or non-treated LPS rats and non-treated control rats. *P<0.05, ** P<0.01 and ***P<0.001 indicates statistical significance (Two-Way ANOVA followed Bonferroni correction) vs. control; #P<0.05 and ##P<0.01 indicates values significantly different (Two-Way ANOVA followed Bonferroni correction) vs. LPS. Values are expressed as the ± SEM (n = 6).

Next, we addressed whether improved anti-inflammatory effects of RVX pre-treatment (5 mg/kg) translate into the amelioration of LPS-induced contractile dysfunction to PE. LPS injection deteriorated PE-induced vasoconstriction when compared with the control group [Emax, 62.33±3.8% for LPS (6 h) compared to controls 97.70.±2.30%, P<0.001; and Emax, 71.91±4.81% for LPS (24 h) compared to controls: Emax, 103.1.±3.61%, P<0.01] (Control *vs* LPS, Emax reduction = 35.4 and 31.19%, P<0.001) **(Fig 4A and 4B) (Table 1).**

**Table 1:** Role of RVX on the vasoreactivity of aortic rings obstained from rats sacrificed 6 h and 24 h post LPS to PE-induced dose dependent contractions.

Contractile Agent	Pretreatment	pEC50	Emax	N
	Control 6 h	7.06±0.06	97.70±2.30	6
PE	LPS 6 h	5.92±0.11+++	62.33±3.80***	6
	LPS+RVX 6 h	6.68±0.06$ $	86.87±2.72##	6
	Control 24 h	6.96±0.06	103.1±3.61	6
	LPS 24 h	6.28±0.12++	71.91±4.8**	6
	LPS+RVX 24 h	6.74±0.08$	91.62±5.8#	6

PE: Phenylephrine. Values are mean ± SEM from (n) different patients. pEC50 and Emax (maximal contraction, % KCl 40 mM) are derived from concentration-response curves presented in Fig 4A and 4B. These values are significantly different:

** *P*<0.01

*** *P*<0.001 *vs* corresponding controls (Control)

# p<0.05

## p<0.01 *vs* corresponding controls (LPS 6 h)

+++ p<0.001

++ p<0.01

$ $ p<0.01

$ p<0.05, when compared to pEC50 values derived from corresponding controls vasoconstriction.

RVX pre-treatment (5 mg/kg) partially reverted LPS-induced PE contractile dysfunction at both 6 h and 24 h following LPS injection (Emax, 86.87±2.72%, and 91.62±5.83%, for LPS+RVX treatment; vs Emax, 62.33±3.8 and 71.91±4.81 for LPS alone, P<0.01), maximal constriction with RVX+LPS was 24.5 and 19.7% higher compared to LPS alone 6 h and 24 h respectively **(Fig 4A and 4B) (Table 1).** These results suggest that RVX attenuates contractile dysfunction to PE during acute LPS inflammation.

## Discussion

In this study, we demonstrated that a specific inhibitor of FXa, namely rivaroxaban, improves acute inflammation and vascular dysfunction following LPS-induced endotoxin shock.

Besides the role of factor Xa in the coagulation process, this factor contributes also to the pathogenesis of cardiovascular inflammatory disease through PARs and non-PAR receptors signaling mediated response in the vasculature [43,44]. Moreover, previous studies have shown that LPS affects the coagulation cascade by targeting FXa and its intracellular signaling which contributed to the increased inflammatory response and vascular modulation mainly through PAR activated receptors signaling [6,45,46]. For instance, the PAR-2 signaling activation contributes to the activation of macrophages and also to vascular inflammation [47]. Interestingly the time-dependent activation of PAR-2 receptors in the vascular and respiratory tissues obtained from rats is induced from LPS itself [48,49]. Also, in other studies, these response was followed by the activation of the inflammatory pathway, via TL-4/NF-κB signaling [50,51]. Most of these responses were shown to be with RVX (Table 2). Of note LPS causes an inflammatory state characterized by increased proinflammatory and pro adhesive responses [52,53], and this could propagate in septic shock and related major complications such as organ failures e.g. respiratory, heart or kidney failures, or abnormal blood clotting (DIC) [54].

**Table 2:** Basic experimental studies that investigate the anti-inflammatory properties of pre-treatment and post-treatment with RVX.

Species	Tissue/Model	Pre-Treatment (1)	Post-Treatment (2)	Response 1	Response 2	References
Rat	Lung/LPS	RVX	-	TNF-α, MCP-1, IL-1β, PAR-2, NF-κB ↓	-	[50]
		0.2 or 0.4 mg/g,				
		10 days				
Rat	Femoral Artery/Atherosclerosis Obliterans	-	RVX	-	IL-1, MCP-1, TNF- α, NF-κB,TLR4 ↓	[51]
			10 mg/kg/day			
			4 weeks			
Rat	Middle Cerebral Artery/Temporary focal cerebral ischaemia	RVX	RVX		IL-1β, IFN-γ, TNF- α, ICAM-1, CD68 ↓	[26]
		3 mg/kg/8 hours	12 mg/kg/ 8 h			
			and 16 h			
Mice	Aorta/ApoE -/-	-	RVX	-	TNF- α, IL-6, MCP-1, Egr-1, IFN-γ ↓	[35]
			1 or 5 mg/kg/day			
			26 weeks			
Mice	Aorta/ApoE -/-	-	RVX	-	TNF- α, COX-2, iNOS, MMP-9, MMP-1 ↓	[14]
			5 mg/kg/day			
			20 weeks			
Mice	Aorta/ApoE -/-	-	RVX	-	PAR-1,PAR-2, Mac-2, MMP-9 ↓	[63]
			1.2 mg/kg/day			
			14 weeks			
Mice	EJV/Catheter		RVX	-	MCP-1, MMP-9 ↓	[15]
	Thrombosis		5 mg/kg/day			
			21 days			
Mice	Atrial/TAC	-	RVX	-	TNF-α, MCP-1, IL-1β, IL-6, PAR-2 ↓	[21]
			30 μg/g/day			
			2 weeks			
Mice	Left Ventricle/TAC		RVX		IL-1β, IL-6, IFN-γ, NF-κB, TGF-β, CD-45 ↓	[77]
			1 or 10 mg/kg/day			
			3 weeks			
Mice	Left Ventricular/Myocardial Ischaemia-RI and TF		RVX		IL-6, PAR-2, collagen	[16]
			0.6 or 1.2 g/kg feed/day		1α2 and 3α1 ↓	
			14 days			
Mice	Heart/Myocardial Infarction	-	138.5mg/kg/day chow		TNF-α, PPAR-1, PAR-2, TGF-β, ↓	[78]
			7 days			
Mice	Aortic root, Coronary Arteries/ICM	-	RVX	-	IL-1β, IL-6, NF-κB	[23]
			10 mg/kg/day		TNF-α, MMP9, MMP12, TIMP1, TGF-β, PAR-1, PAR-2 ↓	
			2 weeks			
Mice	Kidney/Ren-TG Hypertensive	-	RVX	-	TNF-α, MCP-1, Pal-1, PAR-2 ↓	[24]
			6 or 12 mg/kg			
			1 or 4 months			
Mice	Lung/BERK^ss^, vascular Inflammation	-	RVX	-	IL-6, MPO,TAT ↓	[64]
			0.4 mg/g chow			
			10 days			
Mice	Hind Limb/STZ Diabetes, Ischaemia	RVX	RVX	-	Neovascularisation, CD-31, VEGF ↑	[17]
		1 or 3 mg/kg/day	1 or 3 mg/kg/day			
		2 weeks	3 weeks			
Mice	Femoral Arteries/Wire-Mediated Vascular Injury	RVX	RVX	-	TNF-α, MCP-1, IL-1β, (TGF)-β1, SDF-1, GM-CSF ↓	[25]
		5 mg/kg/day	5 mg/kg/day			
		1 week	1 week			
Human	HUVEC/Thrombin	RVX	-	ICAM-1, ELAM-1, IL-8, MCP-1, CXCL1, CXCL2, TF ↓	-	[19]
		0.3–3000 nM				
		30 min				
Human	HUVEC/Inflammation	RVX	-	TNF-α, IL-6, IL-1β, NF-κB ↓	-	[79]
		1000 nM				
		24 hours				
Human	HUVEC/FXa Inflammation	-	RVX	-	CCL-2,CCL-5, EDN2, ITGA5, SELE, VCAM-1, TNSF10, MMP-2 ↓	[80]
			50 nM			
			12 h			
Human	Abdominal Aorta/Aneurysm	-	RVX	-	IL-6, NOS-2, MMP-9 ↓	[20]
			50 nM			
Human	Podocytes/Ang-II-induced Inflammation	RVX	-	-	TNF-α, MCP-1, IL-6, PAR-2, NF-κB ↓	[24]
		500 μg/L				
		1 hour				
Human	Kidney Tubular Cells/AGEs	-	RVX	-	MCP-1, ↓	[62]
			300 nM			
			4 hours			

Abbreviations: RVX, Rivaroxaban; LPS, Lipopolysaccharide; AGEs, Advanced glycation end products; RI, Reperfusion Injury; Ang-II, Angiotensin II: STZ, Streptozocin; BERK^ss^, Berkeley Sicle Cell Mice; Ren-TG, Transgenic Ren-2 Mice;TAC, Transverse Aortic Constriction; ICM,Ischaemic Cardiomyopathy; HUVEC, Human Umbilical Vein Endothelial Cells;TNF-α,Tumour Necrosis Factor Alpha;MCP-1, Monocyte Chemottractant Protein;IL-1β,Interleukin-1 Beta;PAR-2, Protease-Activated Receptor 2;NF-κB, Nuclear Factor Kappa Betta;MMP, Matrix metallopeptidase;GM-CSF,Granulocyte-Macrophage Colony-stimulating Factor; SDF-1,Stromal Cell-Derived Factor 1;VEGF,Vascular Endothelial Growth Factor;NOS-2,Nitric Oxide Synthase-2; Egr-1,Early Growth Response Protein 1;IFN-γ,Interferon Gamma; TF,Thrombin Factor; CCL, C–C Motif Chemokine Ligand, EDN2, Endothelin-2, ITGA5, Integrin Alpha-5/Beta-1; SELE, E-Selectin;TNSF10, Tumor Necrosis Factor (Ligand) Superfamily, Member 10; TLR4, Toll Like Receptor 4.

Nowadays, there are different experimental and clinical therapeutic interventions in sepsis [55–57], and targeting the cross-talk between inflammation and coagulation represents an emerging approach for targeting acute conditions as well as improving long term vascular outcomes in inflamed conditions [8,58,59].

Most clinical studies demonstrated that targeting Factor Xa inhibition with NOAC including rivaroxaban prevented systemic thromboembolic disease, reduced cardiovascular events, and death [60]. Moreover, NOAC non-hemostatic cellular effects suggest a potential benefit in inflammation, arterial stiffness, neointima formation, atherosclerosis, and fibrosis [5,61].

Although some anti-inflammatory effects, improvement of hypercoagulable actions such as disseminated intravascular coagulation (DIC), additional acute lung injury from endotoxemia [19,36,50], and additional vasculoprotective properties of RVX have been proposed in different in vitro and tissue models [14,15,20,21,23–25] **(see Table 2),** a beneficial effect on LPS induced acute vascular inflammatory response in vivo was not investigated yet. In murine macrophages and human tubular cells stimulated with FXa, RVX treatment was shown to reduce the expression of TNF-α, IL-1β, and MCP-1 [14,62]. Similarly, RVX dampened the expression of VCAM-1, ICAM-1, MCP-1, IL-8, CXCL1, CXCL2, TF in thrombin stimulated human endothelial cells [19], as well as IL-6, IL-1β, TNF-α, MMP9, and COL-1 expression in hypoxic cardiac myocytes and fibroblasts [23]. Similarly, also TNF-α, MCP-1, IL-6 expression in angiotensin II-induced inflammatory response in human podocytes was modulated by RVX [24].

Herein, our results extend these findings by demonstrating in vivo that RVX pre-treatment decreased the expression of pro-inflammatory mediators and adhesion molecules namely IL-6, MCP-1, and VCAM-1 induced by LPS in the aorta.

Earlier studies in ApoE-deficient mice showed that chronic administration of RVX reduced gene and protein expression for IL-6, TNF-*α*, MCP-1, i-NOS, COX-2, MMP9 in thoracic and abdominal aortas, attenuated macrophage activation, necrotic core formation, collagen loss, and promoted the stabilization of the atherosclerotic plaque [14,35,63].

Interestingly, a recent study has shown a cardioprotective effect of RVX pre-treatment in ischaemic cardiomyopathy in mice model with diet-induced myocardial infarction [23]. This study has shown that attenuation of cardiac remodeling, fibrosis, alleviation of the aortic root and coronary arteries atherosclerosis is dependent on the reduction of IL-1β, TNF-α, IL-6 cardiac mRNA expression, and nuclear factor kappa B (NF-κB) activation pathway in RVX pre-treated group. Also in the myocardial reperfusion injury mice model, RVX improved survival rates, cardiac function, and reduced IL-6, collagen 1α2, and 3α1 cardiac mRNA expression [16]. RVX protective effects were shown also in a rat model of brain ischemia/reperfusion injury where it reduced VCAM-1 protein expression, macrophage activation, and thrombin mediated thrombus formation [26], and also in pressure overload-induced atrial remodeling with transverse aortic constriction mice model where a reduced macrophage infiltration associated to a decreased expression of MCP-1, IL-6, IL-1β, TNF-α was observed [21].

Moreover, additional studies showed that RVX pre-treatment prevented the development of mechanical femoral vascular injury-induced neointima hyperplasia in mice, again by affecting IL-1β and TNF-α gene expression [25]. Similarly, RVX treatment decreased MCP-1 plasma levels and MMP-9 protein levels in the external jugular vein of mice following catheter thrombosis [15], as well as IL-6 plasma level and neutrophil levels in a mouse model of sickle cell disease [64]. Incubation, ex-vivo, of human abdominal aortic aneurysmal tissues, resulted in the reduction of IL-6 release and NOS-2, MMP9 protein expression [20].

Also, hypertensive renal damage resulted to be ameliorated by RVX chronic pre-treatment of renin overexpressing mice via specifically targeting of TNF-α, MCP-1, and IL-6 [24]. Thus paving the way also to the other newer FXa inhibitor, which recently demonstrated to affect VCAM-1 and ICAM-1 in uremia induced vascular dysfunction [65].

In addition to vascular inflammation, the acute inflammatory response from LPS induces vascular hyporeactivity and hypotension which were shown to be also time-dependent [66,67], thus displaying the highest level of vascular hyperresponsiveness and iNOS expression 6 hours post-exposure to LPS injection [32,68], as observed in our study experimental model. Moreover, FXa has been found to induce hypotension and inflammation response in vascular endothelial cells [43,44], whereas endotoxin activation of FXa and its intracellular signaling have been shown to trigger vascular tone reduction and hypotension [49,69–71]. This effect was shown to depend on factor Xa induced dilation of the rat aorta through the PAR-2 signaling pathway, a contribute pathway which was implicated also in severe hypotension following septic shock [72].

**Fig 5:**
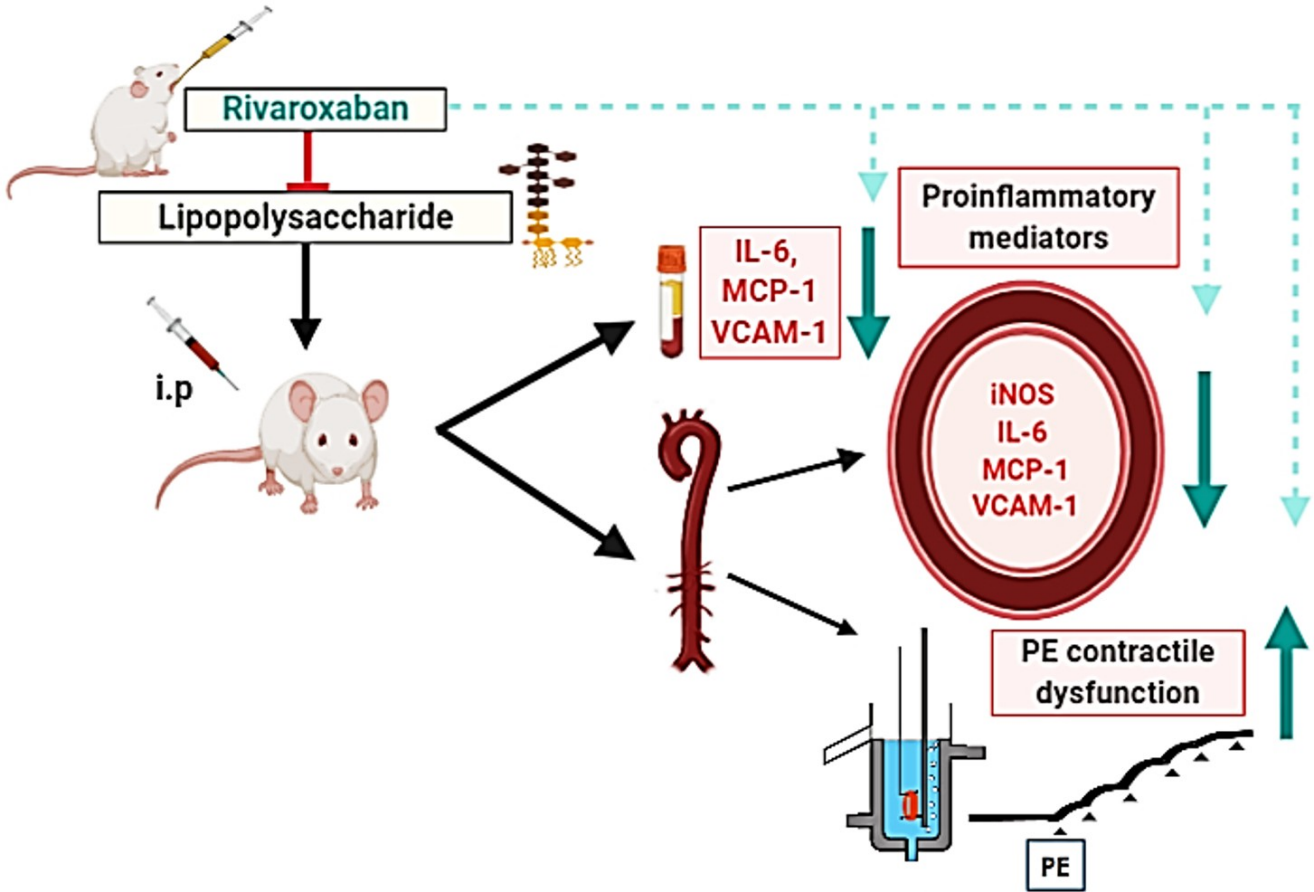
Schematic diagram for the protection of RVX against acute inflammation and vascular dysfunction following LPS-induced endotoxin shock. RVX pretreatment decreased the expression of pro-inflammatory mediators and adhesion molecules and improved aortic hypo responsiveness to PE induced by LPS in the aorta. The figure was prepared with BioRender (biorender.com).

In this study, we demonstrate that RVX pre-treatment improves aortic hyporesponsiveness to PE under inflammatory conditions **(Fig 5)**.

Previous studies tested in vitro the protective role of FXa inhibitors on the vascular tone of control rat aorta [73,74], mesenteric and basilar arteries [31], and streptozotocin-induced diabetic mice [17,75]. We now translate these findings in vivo by showing an improvement of vascular tone in endotoxin-induced hypotension and proinflammatory response following RVX pre-treatment. The effect could rely on the control of FXa-PAR-2 [47–49,69,76] and TL-4 /NF-κB signaling [24,50,51,77].

Although future additional studies are needed to better delineate the mechanisms beyond these effects, the currently available findings set the stage for investigating the additional molecular effects and also clinical benefit of RVX treatment in inflammation and hypotension associated with endotoxin shock.

## Supporting information

S1 Fig Role of RVX pre-treatment on LPS induced proinflammatory and proadhesive mediator’s gene expression in rat liver. Comparisons of relative IL-6, MCP-1, VCAM-1 and ICAM-1 gene expression levels normalized to GAPDH in the rat liver samples obtained from RVX- or non-treated LPS rats and non-treated control rats. *P<0.05, ** P<0.01 and ***P<0.001 indicates values significantly different (Student’s t-test) vs LPS. Values are expressed as the mean±SEM (n = 6).
(TIF)

S1 Table Primer sequences. (DOCX)
