# Supplementary figures and images for: Rivaroxaban improves vascular response in LPS-induced acute inflammation in experimental models
Source: PLoS One. 2020 Dec 10;15(12):e0240669. doi: 10.1371/journal.pone.0240669 (PMC7728205; doi:10.1371/journal.pone.0240669)

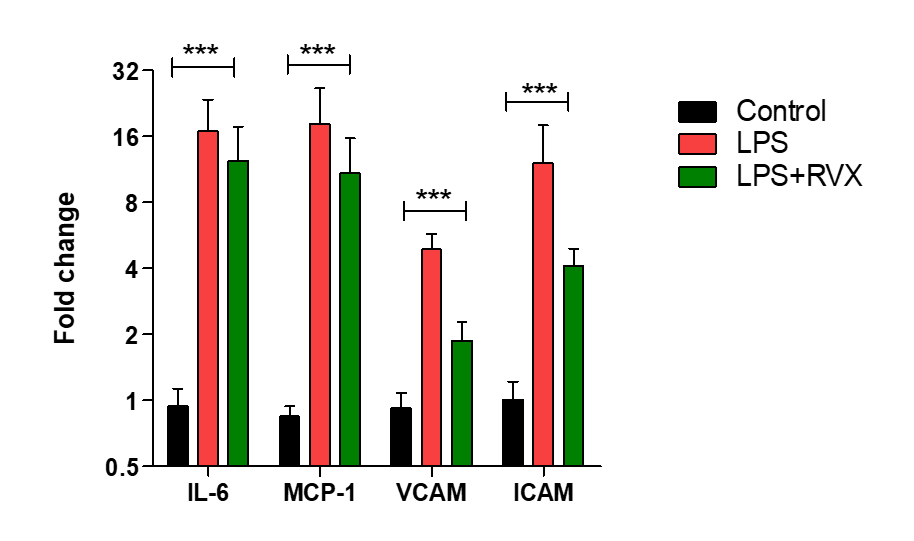

Supplement: S1 Fig — Comparisons of relative IL-6, MCP-1, VCAM-1 and ICAM-1 gene expression levels normalized to GAPDH in the rat liver samples obtained from RVX- or non-treated LPS rats and non-treated control rats. *P<0.05, ** P<0.01 and ***P<0.001 indicates values significantly different (Student’s t-test) vs LPS. Values are expressed as the mean±SEM (n = 6). (TIF) [file pone.0240669.s001.tif]
