# Supplementary material for: Rivaroxaban improves vascular response in LPS-induced acute inflammation in experimental models
Source: PLoS One. 2020 Dec 10;15(12):e0240669. doi: 10.1371/journal.pone.0240669 (PMC7728205; doi:10.1371/journal.pone.0240669)
Supplement: S1 Table — (DOCX) [file pone.0240669.s002.docx]

**S1 Table. Primer sequences**

|  | **Forward** | **Reverse** |
| --- | --- | --- |
| IL-6 | 5′-TTCTCTCCGCAAGAGACTTCC-3′ | 5′-CTCCTCTCCGGACTTGTGAA-3′ |
| MCP-1 | 5′-CCCACTCACCTGCTGCTACTC-3′ | 5′-AGAAGTGCTTGAGGTGGTTGTG-3′ |
| VCAM-1 | 5′-TTTGCAAGAAAAGCCAACATGAAAG-3′ | 5′-TCTCCAACAGTTCAGACGTTAGC-3′ |
| ICAM-1 | 5′-AGATCATACGGGTTTGGGCTTC-3′ | 5′-TATGACTCGTGAAAGAAATCAGCTC-3′ |
| GADPH | 5′-AGGTCGGTGTGAACGGATTTG-3′ | 5′-TGTAGACCATGTAGTTGAGGTCA-3′ |
